# Active digital pedagogies as a substitute for clinical placement during the COVID-19 pandemic: the case of physiotherapy education

**DOI:** 10.1186/s12909-022-03916-4

**Published:** 2022-12-07

**Authors:** Slawomir Wojniusz, Vibeke Dehli Thorkildsen, Silje Therese Heiszter, Yngve Røe

**Affiliations:** Department of Rehabilitation Science and Health Technology, Oslo Metropolitan University, Postboks 4, St. Olavs Plass, Oslo, 0130 Norway

**Keywords:** Clinical placement, Active learning, Collaborative learning, Digital teaching, Physiotherapy

## Abstract

**Background:**

In March 2020, campuses at Norwegian academic institutions were closed due to the COVID-19 pandemic. All in-person teaching had to be replaced by digital alternatives. The closure also affected clinical placements in physiotherapy programs, which in some cases had to be replaced by online alternatives without patient contact. The aim of this study is to evaluate the benefits and challenges of using digital pedagogies to accomplish the learning outcomes of clinical placements.

**Methods:**

Forty-four final-year physiotherapy students at Oslo Metropolitan University had their clinical placement substituted by an online alternative centered around two main educational activities conducted online in small groups: 1) clinical case seminars and 2) digital lectures followed by webinars where students discussed and solved tasks related to the lectures. Additionally, as a part of this alternative placement, students had to conduct a physiotherapy assessment of a family member/housemate and summarize the findings in an anonymized medical record. At the end of the placement, all students wrote a short essay reflecting on their learning process. Students’ written reflections were anonymized and subjected to a qualitative analysis.

**Results:**

Forty-three out of 44 participating students completed their essays. Although students expressed disappointment in missing out on clinical placement, they were surprised by how much learning the online alternative provided. The most valued activities were clinical case seminars where clinical cases previously experienced by the students were discussed. The seminars appeared to facilitate students’ engagement in professional discussions and to enhance their clinical reasoning skills. Seminars also seemed to strengthen students’ belief in their own and their fellow students’ capabilities. Group discussions focusing on topics related to digital lectures were also appreciated. Interestingly, the activity that most closely mimicked a clinical setting – physiotherapy assessment of family member/housemate – was rarely mentioned in the students’ essays. As expected, students most regret not meeting real patients and missing out on the new clinical experiences such encounters would provide.

**Conclusions:**

Despite lack of direct patient contact, students in physiotherapy education evaluated that an online alternative placement was highly clinically relevant. Peer-to-peer discussions of clinical cases appeared to be especially valued. The fact that students themselves had to take the main responsibility for preparing the seminars and leading the discussions was an important pedagogical aspect of the online alternative. The findings indicate that in learning of clinical skills, physiotherapy students take benefit of autonomous, student-centered interventions. Further research should investigate how digital technology-enhanced learning can be used to improve quality of ordinary clinical placement, in physiotherapy- and health education.

**Supplementary information:**

The online version contains supplementary material available at 10.1186/s12909-022-03916-4.

## Background

On Thursday 12 March 2020, the Norwegian authorities locked down the campuses at academic institutions in response to the COVID-19 pandemic. Consequently, all in-person teaching, including clinical placements, had to be replaced with online alternatives. This extraordinary situation forced higher education teachers to rethink the use of educational technology not only in context of traditional teaching but also as a tool in development of practical skills. This article discusses experiences from the Department of Rehabilitation Science and Health Technology, Physiotherapy programme at Oslo Metropolitan University (OsloMet), focusing on student-centered online teaching as a substitute for clinical placement during the pandemic.

Graduation from a physiotherapy programme qualifies the graduate as an independent and autonomous professional [1]. Historically, peer-to-peer practical skills training and clinical placements have been considered essential in development of clinical skills among physiotherapy students. However, new health care reforms such as the Norwegian Collaboration reform [2], compels physiotherapists to perform increasingly more complexed tasks, often in collaboration with other health care professionals. Ability to communicate with stake holders, to function in intra- and inter-disciplinary teams, and user-education became as important as practical clinical skills. In order to facilitate transfer from educational context to actual work practice, teaching methods should reflect the types of tasks that health care professionals are expected to handle. Application of student-active teaching methods [3], and technology-enhanced learning may narrow the gap between educational context and professional work.

Technology-enhanced learning is a term used to describe the application of information and communication technologies to enhance teaching and learning, that is not linked to a specific pedagogical model [4]. During the pandemic, online teaching became a mainstay pedagogical approach. While such teaching can be limited to online streamed lectures, its real benefits lay in the opportunities of integrating the technology with planned, active learning pedagogy. An example of such integration is an implementation of flipped classroom and collaborative learning methods in an online environment. The flipped classroom model implies that digital learning material is provided to students as preparation and that classroom time is used for active teaching and learning [5]. It has been claimed that one of the benefits of the model is the development of student engagement, metacognition, understanding and performance by giving them more control over what and how they learn [6]. Collaborative learning refers to a teaching method in which students work together in small groups towards common goals. This teaching approach seems to stimulate critical thinking, positive interdependence, individual accountability, and to improve interpersonal skills [7]. Compared to competitive and individualistic efforts collaborative learning results typically in higher achievement, greater productivity, self-esteem and social competence [8].

Online learning has in many cases been shown to be superior to in-person teaching in health professions [9–11]. Nevertheless, its implementation in health care education has been slow. Findings suggest that within higher education there is a lack of interventions in which the technology is combined with planned pedagogical learning activities [12, 13]. Moreover, few studies have investigated how digital learning designs can facilitate learning of practical skills and behaviour [11].

The COVID-19 pandemic forced academic staff to rethink educational practices overnight, profoundly accelerating the introduction of digital teaching in higher education. The aim of this study was to evaluate the experiences of students in physiotherapy education participating in a fully online clinical placement. The specific research questions were: 1. What were the benefits and challenges offered by the online alternative placement in terms of achieving learning outcomes? 2. Which elements of the intervention offered the most valuable learning?

## Method

The presented data are a result of a qualitative thematic analysis of the students’ essay assignments. We hereby confirm that all methods were performed in accordance with the relevant guidelines and regulations, following the standards recommended by the NSD—Norwegian Center for Research Data (www.nsd.no/en/) and OsloMet rules for handling and analyzing of research data. Before being subjected to analysis, all the assignments were fully anonymized and original records deleted.

During the third and final year of the physiotherapy program at OsloMet, students must complete two nine-week clinical placements where they treat patients under the supervision of a clinical supervisor. One of the placements is conducted at a secondary care unit, typically a hospital, while the second involves treating patients in a primary care setting, for example an outpatient clinic or a communal rehabilitation center. The clinical supervisors have overall responsibility for treatment of the patients; however, students are encouraged to gradually take more responsibility for planning their clinical activities and patient treatment.

## Learning outcomes

Towards the end of clinical placement students are evaluated according to a set of learning outcomes divided into three categories; *knowledge, skills* and *general competence* (for full list, see Appendix). *Knowledge* category focuses on integration of theoretical knowledge and current research literature into clinical work. *Skills* category centers on student’s ability to plan, conduct and evaluate physiotherapy assessment and treatment. *General competence* covers student’s ability to follow rules and laws, to show appropriate conduct while collaborating with patients and others, and to self-reflect. Online alternative placement was subject to the same set of learning outcomes as a regular placement.

## Online alternative placement

At the time of the COVID-19 lockdown, all third-year physiotherapy students at OsloMet had already conducted at least one of their nine-week clinical placements. Although some placements were still offered, an online alternative that precluded patient contact had to be developed by the department’s academic staff for 44 students. The online alternative placement was planned during a 14-day period and implemented between 27 March and 20 May 2020. In terms of learning outcomes, it was particularly challenging to address those related to skills practiced at the clinical workplace. Furthermore, during their regular placement students are expected to take increasingly greater responsibility for organizing and conducting their clinical activities. These challenges informed the choice of learning activities used as part of the online alternative. Specifically, experience gained from a previously conducted flipped classroom intervention that emphasized self-directed learning and collaborative working was utilized. This model was based on a combination of digital and in-person learning activities [14].

**Fig. 1:**
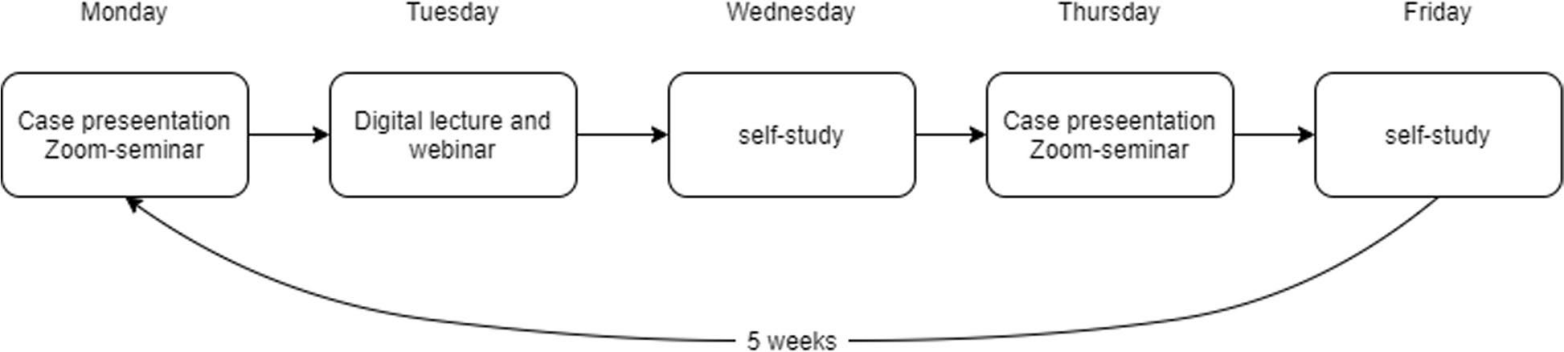
Weekly activities during the online alternative placement. Digital lectures necessary to prepare for webinars were made available for students at least one day before the webinar. In addition to the activities shown in this figure, students conducted a physiotherapy assessment of a family member or housemate and summarized the findings in written records

Teaching activities were conducted digitally using platform Zoom Video Communications, hereafter called Zoom. First, all students completed an online form about their previous clinical placements. Students were then divided into groups of five or six, prioritizing diversity of previous clinical experiences. Each student group was assigned a supervisor responsible for leading group activities. The digital alternative included two main small-group activities: 1) clinical case studies and 2) digital lectures with webinars (Fig. 1). In addition, students were to conduct a physiotherapy assessment of a family member or housemate and to summarize the findings in an anonymized medical record.

## Clinical case studies

Clinical case studies were the main activity of the online alternative placement. The students had to submit two anonymized clinical cases based on experiences from their previous placement. The cases had to be a maximum of 350 words and include topics for group discussion. Notably, the students were encouraged to present cases that they had learned a lot from and which could provide good learning opportunity for their peers. Additionally, students had to recommend relevant sources such as scientific papers or clinical guidelines. The proposed cases were reviewed by a supervisor according to their relevance for learning outcomes.

Case seminars were conducted twice a week over a five-week period. A specific case with supplementary literature was distributed to all the students in the seminar group beforehand. The supervisor organized a Zoom meeting, while the student who provided the case was responsible for leading the seminar. Usually, the students would start with a presentation of their previous clinical placement, followed by the case presentation, discussion and a presentation of the literature. The seminars lasted about two hours, and one or two cases were discussed.

## Digital lectures with webinars

A total of five webinars (one per week) were held, covering topics relevant to clinical practice. Following subjects were discussed: 1) Placebo effect in clinical practice. How to communicate with chronic pain patients? 2) Knee and ankle pain; 3) Treatment of patients with chronic pain; 4) Shoulder pain; 5) Low back pain. The webinars were based on digitalized versions of traditional lectures previously conducted during regular clinical placements. A MP4 video file and a set of topics for further discussions were distributed at least one day in advance by the webinar leader. The students were expected to watch the lecture and reflect on the topics before attending the webinar.

At the beginning of each webinar, students were given an opportunity to ask questions related to the digital lecture. Thereafter, they were randomly divided into groups of four to six students using the “breakout room” function in Zoom. The students discussed the given topics in their groups. They were encouraged to invite the webinar leader into their digital room to further deepen the ongoing discussions. Nevertheless, most of the time the students discussed the topics on their own.

## Physiotherapy assessment of a family member/housemate

The main goal of this activity was to provide an opportunity for a student to directly practice clinical skills relevant for the *Skills* category in learning outcomes. In the course of the alternative online placement, students had to interview and examine a family member/housemate about an ongoing or previous musculoskeletal health condition. Before the assignment was given, all students confirmed by an electronic form that this task would be possible to perform. Based on the clinical interview and physical examination, students had to write an anonymized medical record including their clinical reasoning. For this purpose, the medical record template from the outpatient clinic at OsloMet was used. The anonymized medical record covered: medical history from patient’s perspective, assessment of physical function, functional diagnosis, treatment goal, and treatment plan. The student’s work was reviewed and discussed with the supervisor using Zoom.

## Data collection and analysis

At the end of their final clinical placement, physiotherapy students at OsloMet are asked to reflect in writing on their three-year journey towards becoming a physiotherapist. During the pandemic, students were additionally asked to reflect specifically on their learning process during this particular period (Table 1).

**Table 1:** Text of the assignment which served as a source of the data in this study. The final bullet point (in bold) was specifically added in 2020 to capture students’ experiences with the digital alternative placement

Assignment:
*During the final clinical placement, you are to write an essay (350–500 words) with a particular focus on your development as a physiotherapist*
*You are asked to reflect over the following topics:* *• How are you doing as a therapist? (Here you can write about what you find challenging and/or what you are good at)* *• Experiences and factors during the entire physiotherapy program that have influenced your development as a physiotherapist* ***• Your own learning process during the final clinical placement***

Parts of the text commenting on the online alternative placement were extracted and organized in files. The texts were analyzed using nVivo 12 software. The analysis was conducted according to a stepwise thematic analysis, as presented by Braun and Clarke [15, 16]. Thematic analysis has been recommended as an effective tool to summarize key features of a data set, as it encourages the researcher to take a well-structured approach to handling data to help produce a clear and organized final report [17]. The first steps were undertaken to become familiar with the data and to generate the initial codes. Based on these initial steps, themes were identified, reviewed and named [15]. The first author (SW) and the last author (YR) conducted the analyses separately. Thereafter, it was discussed and adjusted in three Zoom meetings until consensus was reached. Two main themes were identified during the analysis: 1) Benefits and challenges of online alternative placement, and 2) The importance of educational activities for achieving learning objectives. The data included in the first theme was further categorized into three subthemes: online alternative placement – disappointment and surprise; advantages of an online alternative placement; and lack of contact with patients. All co-authors were included in the discussions regarding the results.

## Results

Forty-three out of 44 students commented on the online alternative placement in their written assignments. The length of comments ranged from a short paragraph to a full page. In the following, students’ observations and reflections are presented according to the main themes and subthemes identified during data analysis.

## Main theme 1: Benefits and challenges of online alternative placement

### Online alternative placement – disappointment and surprise

The initial reactions to the substitution of a clinical placement with an online alternative was described by most of the students. They emphasized that previous, regular clinical placement was very important for developing their clinical skills, and they expressed disappointment at and low expectations for the online alternative. Nevertheless, although students would still prefer a regular clinical placement, they were generally surprised by how much they learned during the alternative online practice:

*Student id 2: Originally, I should have had my final clinical placement this term in a hospital. I was very motivated for that and was looking forward to having more experience of different patient categories. That said, I must say I am very surprised by the outcome I got from the alternative digital placement.*

*Student id 12: Although of course I would have preferred a normal clinical placement with real patients, I think the digital solution has worked out surprisingly well.*

Although the online alternative precluded contact with patients, it still seemed to facilitate development of skills important for clinical work:

*Student id 11: All in all, I think this has been a valuable learning process, where I have been challenged in many different areas. I also feel that I have developed as a therapist within the various learning outcomes that are included in the study program.*

### Advantages of an online alternative placement

While students expressed that an online alternative could not replace a clinical placement, in some aspects it had advantages over regular placements. In particular, the exposure to a wide variety of clinical cases was valued:

*Student id 34: We have gained experience in a number of different clinical cases and ethical dilemmas, probably with more variety than what we would have had in a normal clinical placement.*

*Student id 18: The students in my group had quite different previous clinical experiences, which broadened our perspective. The opportunity for discussing and asking questions provided me with broader knowledge that I find useful.*

*Student id 13: The last clinical placement has given us [students] the possibility of learning more about each other’s experiences. This has given me the opportunity to gain broader knowledge compared with a different [normal] placement.*

### Lack of contact with patients

The lack of direct contact with patients was recognized as the main challenge:

*Student id 43: We lose the experience in communicating with the patients, not least in getting it ‘under our skin’ and ‘in our fingers’.*

*Student id 15: Guidelines for different diagnoses are important, but at the same time it is important to have experience in adapting the treatment to the individual, focusing on what is important to them.*

## Main Theme 2: The importance of educational activities for achieving learning objectives

The main challenge of online alternative was to accomplish learning outcomes related to practical clinical work. Clinical case seminars and physiotherapy assessment of family member/housemate were meant to address this challenge. Students seemed especially engaged in clinical case seminars; 29 students made comments about this learning activity in their essays. Small-group discussions focusing on clinical cases seemed to be especially valued:

*Student id 40: Working in small groups with clinical cases has been very useful, even without patient contact. It has been organized in a way that facilitates good discussions and variation in the clinical cases.*

*Student id 2: It has been an extremely efficient way of sharing experiences. I have developed my ability to reflect and reason about different clinical cases on the basis of professional discussions with my fellow students.*

Group discussions were perceived as informative, and seemed to reinforce students’ self-belief and confidence in their own and their peers’ abilities:

*Student id 11: Personally, I must say that these discussions of the clinical cases have been very informative. I think that discussing, and hearing different opinions, where you are not afraid to speak out, has been a very good way of getting new thoughts and ideas about the profession. At the same time, I feel I have mastered the situation where we gave lectures about our clinical cases, and have become more self-confident.*

*Student id 22: I have also been impressed and pleasantly surprised by my fellow students. It’s good to hear that others think that the subject can be difficult, but also that they have clinically examined, talked to and seen the patients and what they need help with. To feel that I would have liked to send my loved ones to them as therapists is a good feeling, and makes me proud of my profession.*

On the other hand, physiotherapy assessment of family member or housemate was commented only by four students. Two of the students pointed out that writing a medical record is an important exercise that provides good training in this skill:

*Student id 25: The writing of patient records must also be mentioned. It was good to have a repetition on how to write a medical record.*

Nevertheless, this activity could also be experienced as artificial:

*Student id 11: I felt that this was a bit artificial, but was a nice situation to practice taking a patient history and examination,*

One of the students found this assessment of family member problematic because it challenged previously established boundaries between private and professional life:

*Student id 30: After three years of study I have created a boundary between my private and professional life, where I avoid treating my close ones. And even if just for a short period of time, mixing those roles was demanding.*

Digital lectures with webinars were commented by 13 students. The main goal of this activity was to extend students’ theoretical knowledge and ease its integration into clinical work. Especially group discussions around topics assigned to specific lectures seemed to be valued:

*Student id 25: The joint webinars were extremely educational. The lectures were good, and the assignments and the clinical cases we prepared for meant that we had to reflect on what was presented in the lectures. The discussions about the assignments in the smaller groups were my favorite part of the alternative placement.*

### Challenges of working online

Challenges experienced during online activities were mostly related to their digital format, such as difficulty with keeping discussions going in Zoom or students not willing to have the camera turned on:

*Student id 19: There are of course things that have been annoying, including an unwillingness to have the camera turned on during the different webinars and the case studies. This often leads to difficulties when it comes to creating/starting a discussion,*

Some students also missed meeting their peers in classes and study groups:

*Student id 38: … I have also missed meeting fellow students and discussing and practicing in study groups.*

## Discussion

The substitution, caused by the pandemic, of clinical placement with an online alternative represented in educational terms a dramatic change. While not optimal, this unique situation provided an opportunity to explore alternative ways of achieving learning outcomes typically associated with practical clinical work. In the following discussion, we concentrate on the two main questions: 1. What were the benefits and challenges offered by the online alternative placement in terms of achieving learning outcomes? 2. Which elements of the intervention offered the most valuable learning?

## Achieving learning outcomes

It is not surprising that students felt disappointment when clinical placements were cancelled due to the COVID-19 pandemic. Despite this, their evaluations indicate that the online alternative provided valuable experience. In particular, the variety of clinical cases discussed was emphasized by the students. Some even remarked that the online alternative gave a broader learning experience than regular clinical placements. Regular placements can differ considerably from each other, and students sometimes question whether their specific placement is an optimal learning arena in terms of subsequent exams or their future professional career. Ensuring that students in a seminar group had varied clinical experiences from previous placements, led to greater diversity in the discussed topics and cases than a normal clinical placement would provide. Digital lectures with follow-on webinars provided the opportunity to further broaden learning areas.

Overall, online placement appeared to adequately address learning outcomes requiring the integration of theoretical knowledge in a clinical setting, professional self-reflection, and clinical reasoning. For this purpose, the use of active pedagogy seemed to play a decisive role. Student-active pedagogies such as collaborative learning and flipped class room, allow students to better reflect on their own learning process as clinicians (meta cognition), in an autonomous manner [18]. It has been argued that student-centred learning is about spaces that provide students with the opportunity to act upon their learning needs, intentions, and interests, thus increasing autonomy in the learning [19, 20]. Previous findings suggest that under-graduate physiotherapy students, if given the opportunity, are able to utilize considerable amounts of autonomy, to the benefit of their learning [14].

Students’ essays indicate that leading the seminars, observing peers in the same role and engaging in peer-to-peer discussions seems to strengthen their self -belief, professional identity and pride. Students also expressed increased confidence in their own clinical capabilities as well as those of their peers. Taking responsibility, initiative and leadership in solving tasks corresponds especially with *General competence* category of learning outcomes in clinical placement. These capabilities are also considered important features in the development of occupational competence [1].

As expected, an online alternative placement showed to be inadequate in terms of exercising practical skills. The lack of face-to-face interaction with patients was reported as its main drawback. Indeed, practicing clinical skills and developing a therapeutic alliance, is impossible to re-create fully in online education. To address this shortcoming, the students were to conduct a clinical examination of a family member or housemate. The practice of assessment procedures and writing medical records were commented on as useful, but could also be perceived as artificial. In addition, one of the students raised ethical concerns with this task. The student had previously avoided practicing clinical skills with family members and found it demanding to cross the line between private and professional life. This is an important ethical issue to consider when planning practical exercises. Although, all students had first confirmed by an online form that this task would be possible to complete, it still placed one of them in a difficult position. In the future, ethical challenges associated with practicing clinical assessment or treatment of close ones should be thematized beforehand [21] and alternative solutions should be planned. Since only four students commented on this part of the online alternative, we cannot be certain of the students’ overall experience. Nevertheless, it is possible that making this learning activity collaborative would result in higher student engagement and improved learning outcome. In small groups or pairs, students could discuss their clinical evaluations with each other, design treatment plans and give feedback on anonymous medical records.

## Collaborative learning

Clinical case seminars seemed to be the most valued part of online alternative. They were designed around the principles of collaborative learning [22]. According to Roger & Johnson (1994), in order for collaborative learning to be successful it needs to include five elements; positive interdependence, considerable interaction, personal responsibility and accountability, frequent use of social skills and group self-evaluating [23]. Personal responsibility and considerable interaction were especially prominent aspects of case seminars. Every member of the seminar group had responsibility for preparing and leading two seminars. During a seminar, the presenting student had to take on the role of the expert in the particular clinical case and lead a discussion centered on specific topics. No other seminar participants, including the supervisor, had any prior information about the case, which may have further empowered students to take leadership roles and to take part in the discussion. After concluded seminar, the group discussed improvements to its content and format. Self- evaluation is an important feature of collaborative learning [22] and it helped to ensure continuous improvement of the seminars quality.

In another learning activity, the webinars, students were randomly assigned to a new group at each webinar. This is against one of collaborative learning principals, recommending that students work in the same group over longer period of time [23]. In this way they can easier develop and practice trust-building, leadership, decision-making, communication, and conflict management skills [22]. This difference in small-group design was not commented by the students but it is possible that permanent groups would further improve learning outcome of webinars.

In contrast to case seminars, group discussions during webinars were conducted without the presence of a supervisor. Nevertheless, the differences in supervisor roles between the case seminars and the webinars, were not commented by the students. Instead, their comments show that they consistently underscore the central role of peer-to-peer collaboration during both activities. These findings are rather similar to previous experiences in technology-enhanced learning, at our department [14]. This suggests that having the supervisor present during the group discussion is not a necessary precondition for the group's success.

## Strengths and limitations

The findings of this study are based on students’ written essays, and represent their spontaneous reflections on the learning process during an online alternative placement. As such, their statements could not be followed up by supplementary questions, as would, for example, be natural during an individual or the focus group interview. This may give a somewhat fragmented picture of experiences with the online alternative and leave many potentially interesting questions unanswered. The strength of the study is that it provides quite a comprehensive picture of the students’ experiences, as 43 out of 44 participating students commented on the online alternative in their essays.

## Implications

Technology-enhanced learning can never fully replace face to face, clinical education. However, we hope that findings from this study will stimulate to rethink ordinary practice placements in physiotherapy and health education. Integration of collaborative learning activities into clinical placements can potentially improve achieving learning outcomes related to clinical reasoning, self-reflection and leadership skills. We also believe that the largely positive experiences with an active online practice should be used to further develop pedagogical activities aimed at improving students’ practical skills.

## Conclusions

Despite being developed in the course of merely two weeks, an online alternative placement seemed to provide valuable learning and experiences. Peer-to-peer discussions of a wide variety of clinical cases taken from different practice arenas appeared to be especially valued. The fact that students had to take main responsibility for preparing and leading the seminars was an important pedagogical aspect of the online alternative. The present findings support the implementation of active, collaborative learning pedagogies in practice placement. Future research should investigate how these pedagogical elements can be implemented in ordinary practice placement, and how technology-enhanced learning can improve future education in this field.

## Supplementary Information

**Additional file 1.** 

## Data Availability

Original, anonymized data (in Norwegian language) are available after request to corresponding author.

## Abbreviations

OsloMet: Oslo Metropolitan University
Oslo: Norway
