# Supplementary material for: Active digital pedagogies as a substitute for clinical placement during the COVID-19 pandemic: the case of physiotherapy education
Source: BMC Med Educ. 2022 Dec 7;22:843. doi: 10.1186/s12909-022-03916-4 (PMC9727907; doi:10.1186/s12909-022-03916-4)
Supplement: Supplementary file 1 — Additional file 1. [file 12909_2022_3916_MOESM1_ESM.docx]

Appendix – learning outcomes

Knowledge

The candidate can

1. Integrate previously acquired theoretical knowledge in clinical work and discuss it in light of current theory
2. Acquire, evaluate and apply relevant research literature in clinical work

Skills

The candidate can:

1. Choose, apply and justify physiotherapy assessment methods in light of International Classification of Function framework
2. Identify signs of possible worrisome, pathological conditions
3. Identify environmental and personal factors and evaluate their impact on patient’s function, working ability and health
4. Interpret and summarize findings from the physiotherapy assessment and provide functional diagnosis based in a biopsychosocial perspective
5. Establish treatment goals in collaboration with patient
6. Plan and conduct physiotherapy treatment, evaluate its progress and apply relevant adjustments to treatment plan
7. Plan and conduct preventive measures relevant for patient’s condition
8. Enable patient to increase its knowledge and to apply it in daily living
9. Show empathy, stimulate patient involvement and establish relationship based on equality
10. Communicate and collaborate with patient and others; evaluate need for interdisciplinary collaboration
11. Document relevant information in medical journal and provide clinical reasoning supported by current literature

General competence

The candidate can:

1. Follow current laws, rules and ethical guidelines for physiotherapists, e.g. with regard to confidentiality, privacy protection etc.
2. Follow specific rules of clinical placement with respect to organising your own work, following procedures for medical record documentation, keeping order and appropriate hygiene standards
3. Show respect for patient’s and others points of view and wishes, and for cultural and social differences
4. Reflect over own professional conduct, identify learning needs and take initiative and responsibility to acquire new knowledge and skills
5. Show willingness and ability to give constructive feedback and to make use of supervision

| Learning outcomes | Pedagogical activity |
| --- | --- |
| Knowledge | |
| 1. Integrate previously acquired theoretical knowledge in clinical work and discuss it in light of current theory | All |
| 1. Acquire, evaluate and apply relevant research literature in clinical work | PhA, CCS |
| Skills | |
| 1. Choose, apply and justify physiotherapy assessment methods in light of International Classification of Function framework | All |
| 1. Identify signs of possible worrisome, pathological conditions | All |
| 1. Identify environmental and personal factors and evaluate their impact on patient’s function, working ability and health | PhA, Web |
| 1. Interpret and summarize findings from the physiotherapy assessment and provide functional diagnosis based in a biopsychosocial perspective | PhA |
| 1. Establish treatment goals in collaboration with patient | PhA |
| 1. Plan and conduct physiotherapy treatment, evaluate its progress and apply relevant adjustments to treatment plan | CCS, Web |
| 1. Plan and conduct preventive measures relevant for patient’s condition | CCS |
| 1. Enable patient to increase its knowledge and to apply it in daily living | CCS |
| 1. Show empathy, stimulate patient involvement and establish relationship based on equality | CCS |
| 1. Communicate and collaborate with patient and others; evaluate need for interdisciplinary collaboration | PhA |
| 1. Document relevant information in medical journal and provide clinical reasoning supported by current literature | PhA |
| General competence | |
| 1. Follow current laws, rules and ethical guidelines for physiotherapists, e.g. with regard to confidentiality, privacy protection etc. | PhA |
| 1. Follow specific rules of clinical placement with respect to organising your own work, following procedures for medical record documentation, keeping order and appropriate hygiene standards | All |
| 1. Show respect for patient’s and others points of view and wishes, and for cultural and social differences | CCS |
| 1. Reflect over own professional conduct, identify learning needs and take initiative and responsibility to acquire new knowledge and skills | All |
| 1. Show willingness and ability to give constructive feedback and to make use of supervision | All |
